# CPEB3 can regulate seizure susceptibility by inhibiting the transcriptional activity of STAT3 on NMDARs expression

**DOI:** 10.1186/s10020-025-01136-2

**Published:** 2025-02-24

**Authors:** Zhipeng You, Cong Huang, Fan Wei, Jiran Li, Yang Liu, Xingan Liu, Zhijie Fan, Xiaoying Gao, Jiahang Sun

**Affiliations:** 1 Department of Neurosurgery, The Second Affiliated Hospital of Harbin Medical University, No. 246, Xuefu Road, Nangang District, Harbin, Heilongjiang, 150001 China; 2 Department of Anesthesiology, The Fourth Affiliated Hospital of Harbin Medical University, No. 37, Yiyuan Road, Nangang District, Harbin, Heilongjiang, 150001 China

**Keywords:** Epilepsy, CPEB3, STAT3, Transcription factor, NMDARs

## Abstract

**Background:**

The pathogenesis of epilepsy is complex, and current antiepileptic drugs do not effectively control the seizures. Cytoplasmic polyadenylation element-binding protein 3 (CPEB3) regulates neuronal excitability, but its mechanism of action in epilepsy is not clear. In this paper, we investigated the effect of CPEB3 on seizures and elucidated its underlying molecular mechanism.

**Methods:**

Bioinformatics-based search for genes closely associated with epilepsy. Changes in expression and cellular localization of CPEB3 in epilepsy were verified by western blotting (WB) and Immunofluorescence staining. Subsequently, The adeno-associated virus was employed to overexpress or knockdown in mice. Behavioral experiments verified the effect of CPEB3 on epileptic phenotype, and the molecular mechanism of CPEB3 affecting epileptic phenotype was explored by WB, real-time quantitative polymerase chain reaction (RT-qPCR), RNA immunoprecipitation (RIP), and chromatin immunoprecipitation (CHIP).

**Results:**

The results were that CPEB3 was downregulated epilepsy in model mice and patients with temporal lobe epilepsy and co-expressed with neurons. Behavioral experiments have shown that CPEB3 negatively regulates epilepsy phenotype in mice. In addition, exogenous CPEB3 can also bind to the mRNA of signal transducer and activator of transcription 3 (STAT3) and inhibit its translation, resulting in lower levels of STAT3 and p-STAT3, reduced nuclear translocation of STAT3, and decreased STAT3-mediated transcriptional activity of GluN1, GluN2A, and GluN2B, suppressing the expression of NMDAR subunits, which attenuate the seizure degree and susceptibility of epileptic mice.

**Conclusion:**

These findings suggest that CPEB3 may influence excitability and susceptibility in epileptic mice by regulating STAT3 translation and transcriptional activities to promote NMDARs expression. This mechanism could offer insights into novel therapeutic targets for epilepsy.

**Supplementary Information:**

The online version contains supplementary material available at 10.1186/s10020-025-01136-2.

## Background

Epilepsy is a complex neurological disorder characterized by spontaneous recurrent seizures (Devinsky et al. 2018; Gan et al. 2021). Approximately 70 million people worldwide suffer from epilepsy of varying degrees (Thijs et al. 2019). Patients with epilepsy suffer severe physical and psychological consequences, which further burden families and society financially (Shlobin et al. 2022). Although more than a dozen antiepileptic drugs commonly used in clinics can control the symptoms of epilepsy, 30% of patients still show drug resistance, which is most common in temporal lobe epilepsy (TLE) (Qin et al. 2022). Currently, there is a lack of an effective drug to inhibit the onset or progression of epilepsy. Therefore, exploring new effective therapeutic targets for TLE is urgently needed.

RNA-binding proteins (RBPs) are important proteins that interact with RNA targets and are widely involved in various post-transcriptional regulatory processes, including splicing, localization, degradation, modification, and translation of cellular RNAs, thereby regulating specific gene expression (Liu et al. 2020; Xiao et al. 2024; Zhou et al. 2024). The cytoplasmic polyadenylation element-binding proteins (CPEBs) are a key family of RBPs (Chao et al. 2013; Mendez et al. 2001). Four family members of CPEB have been identified: CPEB1, CPEB2, CPEB3, and CPEB4. CPEB3 is abundantly expressed in the central nervous system (Huang et al. 2006; Morgan et al. 2010; Theis et al. 2003). Studies have shown that CPEB3 regulates neuron-specific selective splicing and neurogenesis gene expression (Qu et al. 2020a, b). In the current study, CPEB3 can mediate the alteration in neuronal excitatory (Hwang et al. 2022). Altered neural excitability is an important factor contributing to seizures (Casillas-Espinosa et al. 2012; Ke et al. 2023). This suggests that CPEB3 may be closely related to epilepsy.

Activation of the Janus kinase/signal transducer and activator of transcription (JAK/STAT) pathway is observed in several central nervous system disorders, including epilepsy (Han et al. 2020; He et al. 2020; Hristova et al. 2016; Li et al. 2021; Toral-Rios et al. 2020). Research has demonstrated that the JAK/STAT pathway is involved in epileptic progression and remodeling of synaptic plasticity in hippocampal neurons (Nicolas et al. 2012). Activator of signal transducer and activator of transcription 3 (STAT3) is a key factor in the JAK/STAT pathway. It is also a member of the mammalian signal converter and activator of the transcription family that plays an important role in signal transduction (Kisseleva et al. 2002; Li et al. 2024; Xu et al. 2023). The results of animal experiments show that inhibition of STAT3 activation reduces seizure severity and delays seizure progression in epileptic mice (Martín-Suárez et al. 2023). It has been reported that activation of STAT3 is regulated by CPEB3 (Fang et al. 2020). However, it is not clear whether this mechanism exists in epilepsy.

In this study, we investigated a potential mechanism by which CPEB3 influence TLE. Bioinformatics analyses initially suggested that CPEB3 could be associated with epilepsy, and further experiments confirmed that CPEB3 expression was reduced in both epilepsy mouse models and patients with TLE. Our findings indicate that CPEB3 modulate seizure severity and susceptibility in epileptic mice. Mechanism studies suggest that exogenous CPEB3 binds to STAT3 mRNA and inhibit its translation, which could alter STAT3 nuclear translocation and reduce STAT3-mediated transcription of GluN1, GluN2A, and GluN2B, ultimately downregulating NMDAR expression. These findings offer insights into the potential role of CPEB3 in epilepsy and highlight it as a prospective therapeutic target.

## Materials and methods

### Human brain tissue

This study was approved by the Ethics Committee of the Second Hospital of Harbin Medical University and was conducted per the Declaration of Helsinki (Approval Number: KY2024-036). In this study, the human brain tissue samples were collected from the Second Hospital of Harbin Medical University, and written informed consent was obtained from the patients. Tissue samples were collected from 12 patients. These tissues were obtained from patients with TLE and patients with traumatic brain injury (TBI). All tissue specimens were stored in liquid nitrogen or 4% paraformaldehyde solution immediately after sampling until use. The clinical characteristics of the patients were shown in Table S1.

### Animals

In this study, 6–8 weeks C57BL/6J male mice were purchased from the Animal Laboratory Center of the Second Affiliated Hospital of Harbin Medical University. The mice were randomly assigned to the experimental group. All mice were kept in a constant temperature and humidity environment. All animal experiments were approved by the Ethics Committee Second Affiliated Hospital of Harbin Medical University (Approval Number: KY2018-108).

### Bioinformatics analysis

Briefly, bioinformatics analysis includes Gene Expression Omnibus (GEO) dataset collection, extraction of RBPs-related genes, differential expression analysis, screening of Hub genes, and construction of diagnostic model and single-cell analysis. The detailed experimental steps can be seen Supplementary document.

### Kainic acid (KA) model

Mice were anesthetized with 4% sodium pentobarbital. Following general anesthesia, mice were positioned in a stereotaxic apparatus and the hippocampal region was injected with 0.6 µL of KA (0.5 µg/µL) (Sigma, USA).The specific coordinates of the bregma origin were: anteroposterior (AP): -2.0 mm; mediolateral (ML): ±1.5 mm; dorsoventral (DV): -2.0 mm (Gao et al. 2023). The amount of saline given to the control mice was the same. The needle was inserted and held in for 5 min before being gradually removed. Seizure scores were evaluated using the Racine scale (Racine 1972). A score of IV and higher was considered successful modeling.

We induced an acute seizure model using intraperitoneal injection of of KA (25 mg/kg) (Liu et al. 2022)The time of the first generalized seizure (stages IV and V) in mice was recorded after KA injection was regarded as the epileptic latency.

### Pentylenetetrazole (PTZ) model

PTZ was injected intraperitoneally into the mice. Seizures were induced from repeated intraperitoneal injections of a subconvulsive dose (35 mg/kg) of PTZ (Sigma, USA) every two days (Yu et al. 2021; Zhang et al. 2022). Each injection was followed by a half-hour of animal observation. Throughout the trial, seizures were tracked and assessed using the Racine scale.

### Adeno-associated virus (AAV) injection

Overexpression of CPEB3 virus was purchased from Obio Biotechnology Co. (Shanghai, China). For overexpression of CPEB3 pAAV-CMV-MCS-3xFLAG-WPRE was utilised as a vector. Knockdown CPEB3 virus was purchased from the Shanghai Genechem. (Shanghai, China) For knockdown of CPEB3 was achieved by CPEB3-RNAi piggybacking pAAV-U6 -CAG-WPRE as a vector. The titers used were of overexpression virus were 3.49 × 10^13^ vg/ml and 2.87 × 10^13^ vg/ml for knockdown virus.

A 1 ul microsyringe was used to inject viruses into the mouse CA1 brain area stereotactically. The specific coordinates of the bregma origin were: AP: -2.0 mm; ML: ±1.5 mm; DV: -1.7 mm. The injections were performed bilaterally at a dose of 0.5 ul per side and a rate of 0.2 ul/min; the needle was kept in place for 5 min after completion of the injections. KA or PTZ-induced epilepsy was performed three weeks after the virus injection.

### Western blotting (WB)

Brain tissue samples were collected for WB analyses. Total protein was extracted using RIPA buffer (Beyotime, China), a protease inhibitor (Beyotime, China), and a phosphatase inhibitor (Biosharp, China). The other components follow the manufacturer’s instructions (cytoplasmic and nuclear proteins: Thermo Fisher, MA, USA; membrane proteins: Beyotime, China; synaptosome proteins: Invitrogen, CA, USA). Initially, protein samples were separated using sodium dodecyl SDS-polyacrylamide gel and electrotransferred onto a PVDF membrane (Millipore, MA, USA). After that, the membranes were sealed for two hours, and the primary antibody was then incubated at 4 °C for the entire night. Finally, the secondary antibody was incubated at room temperature for 2 h, followed by rinsing with TBST, and the blots were visualized using an ECL reagent (Epizyme, China). Specific antibody information is shown in Table S2.

### Immunofluorescence staining

After deparaffinization, the brain tissue pieces were submerged in a 1× citrate solution and heated for 10 min under high pressure. Triton X-100 (Beyotime, China) permeabilized the cell membranes for 15 min. The goat serum working solution (Boster, China) was blocked at 37 °C for 30 min. Then, the primary antibody was incubated overnight at 4 °C, and a secondary antibody corresponding to the primary antibody was used. DAPI has stained the nucleus (Beyotime, China) and photographed under a fluorescence microscope (Nikon, Japan). Specific antibody information is shown in Table S2.

### Local field potentials (LTP) recording

Briefly, two screws were implanted into the mouse skull as grounding screws, and electrodes (Kedou, China) were subsequently implanted into the CA1 brain region of mice using a stereotactic apparatus (Tang et al. 2023). 2 h LFP signals were recorded and data were analyzed using NeuroExplorer (Nex Technologies, MA, USA).

### Drug administration

WP1066 (MCE, NJ, USA) (a STAT3 inhibitor) was dissolved in dimethyl sulfoxide (DMSO). Knockdown CPEB3 mice were injected intraperitoneally with 40 mg/kg WP1066. Equal volumes of DMSO were administered to control animals. Intraperitoneal injection once daily for 7 days before induction of KA or PTZ.

### Real-time quantitative polymerase chain reaction (RT-qPCR)

Total RNA was extracted from mouse hippocampal tissues using the TRIzol reagent (Invitrogen, USA). To get cDNA, the manufacturer’s (TransGen Biotech, China) instructions were followed. The qPCR procedure was carried out using the PerfectStart^®^ Green qPCR SuperMix kit (TransGen Biotech, China). Primer sequences are shown in the Table S3.

### RNA Immunoprecipitation (RIP)

An RNA immunoprecipitation kit (Geneseed, China) was used to perform RIP. Briefly, fresh hippocampal tissue from mice overexpressing CPEB3 was lysed. Immunoprecipitation was performed using an anti-FLAG antibody. IgG was used as a control. Subsequently, RT-qPCR was used for quantitative analysis. The primer sequences are shown in Table S3.

### Chromatin Immunoprecipitation (CHIP)

CHIP was performed according to the instructions of the Sonication CHIP Kit (Abclonal, China). In summary, fresh hippocampal tissues were crosslinked using 1% formaldehyde, followed by nucleus extraction, chromatin sonication and fragmentation, and co-incubation with sonication lysate using an anti-STAT3 antibody. The chromatin was eluted and de-crosslinked. Finally, DNA was purified using a DNA purification kit (Abclonal, China). RT-qPCR was performed to quantitatively analyze the experimental results. The primer sequences are shown in Table S3.

### Statistical analysis

The data were analyzed with GraphPad Prism (version 8.0) and given as mean ± standard deviation (SD). Student’s t-test was utilized for comparisons between two groups.Tukey’s post-hoc test was used in conjunction with a one-way or two ANOVA for multiple comparisons. Statistical significance was set at *p* < 0.05. Non-significant differences were designated as “ns” (*p* ≥ 0.05), whereas ****p < 0.0001, ***p < 0.001, **p < 0.01, and *p < 0.05 represent significant differences.

## Results

### Bioinformatics results of RBPs-associated gene expression profiles in TLE patients

**Fig. 1:**
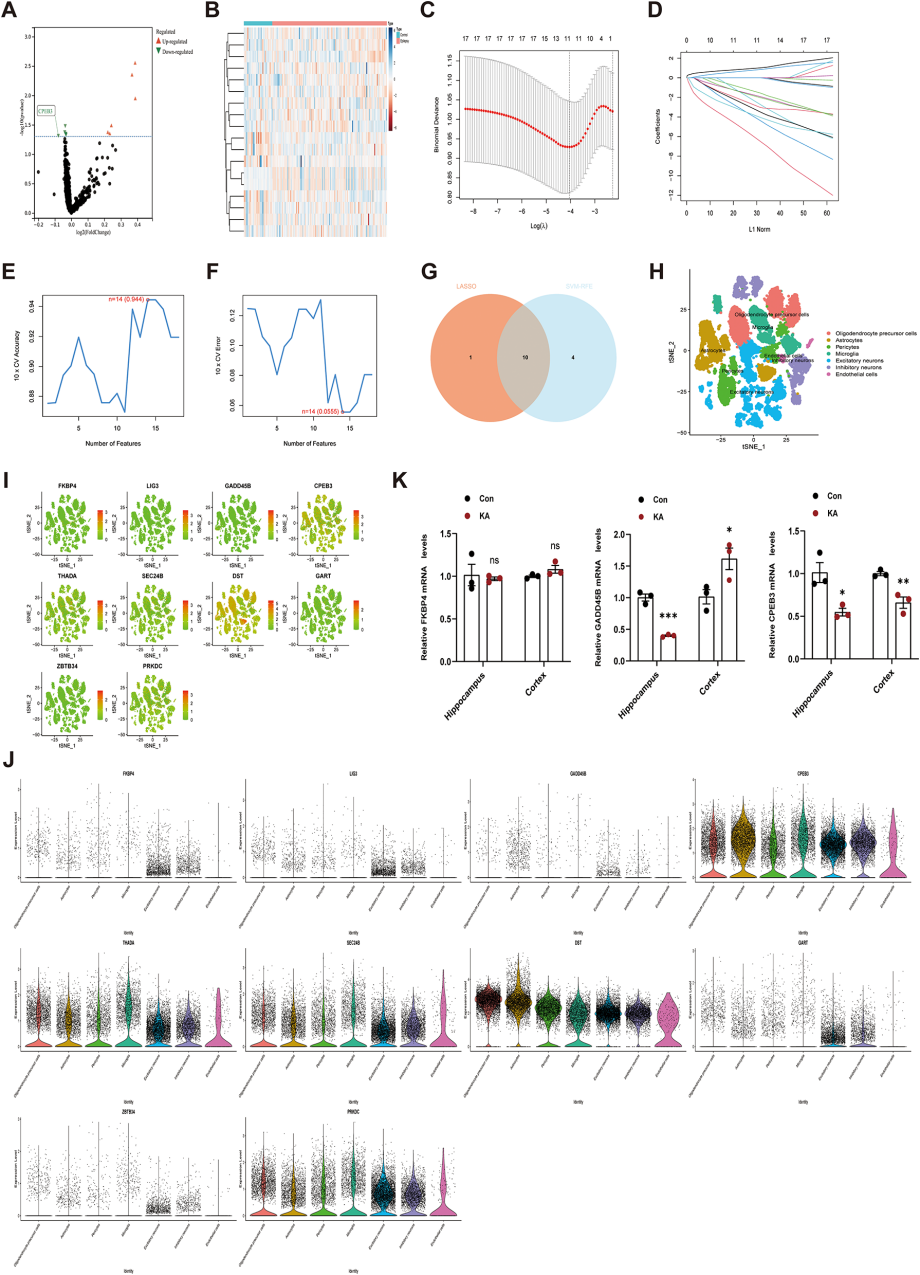
Bioinformatics results of RBPs-associated gene expression profiles in TLE patients. **A** The volcano graphic displays the differential expression analysis results. The y-axis shows -log10 (*p*-value), and the x-axis log2 (Fold Change). Black dots show genes with no discernible differential expression, red triangles represent down-regulated genes, and green triangles represent up-regulated genes. **B** Gene differential heatmap. The graph’s columns stand for samples, its rows for genes, and the colors blue and red correspond to the gene’s expression level, which ranges from high to low. The blue and red colors at the top of the heatmap stand for the Control and Epilepsy groups. **C**,** D** Screening for Hub genes using the LASSO logistic regression. **E**,** F** The Hub genes were screened by the SVM-RFE algorithm for feature selection. **G** The Hub genes shared by the LASSO and SVM-RFE methods are displayed in the Venn diagram. **H** The tSNE dimensionality reduction algorithm was used to group the cells into seven cell types, with each color denoting a phenotype that was annotated for each cluster. **I**,** J** The distribution of each Hub gene in various cell types is displayed via the feature map and violin plots. **K** The mRNA level of FKBP4, GADD45B, and CPEB3 mRNA in the hippocampal cortex of mice with KA-induced epilepsy. (*n* = 3)

We assessed the batch effects before and after merging GSE63808 and GSE29738. After removing the batch effects, the data suggested that the batch effect was better removed from the merged dataset. The dataset was used for subsequent analyses (Fig. S1). Subsequently, we performed a differential expression analysis of the merged and extracted RBPs-associated gene expression profiles. We identified 18 RBPs-associated differentially expressed genes, including 6 upregulated and 12 downregulated genes (Table S4). Notably, the gene of CPEB3 was downregulated in epilepsy patients (Fig. 1A, B). Furthermore, we explored the possible signaling pathways involved in 18 differentially expressed genes using GO and KEGG enrichment analysis (Fig. S2). We used two algorithms to screen for potential Hub genes in the TLE. The 18 RBPs-associated differential genes listed above were reduced using the LASSO regression technique, resulting in the identification of the 11 differentially expressed genes most strongly linked to TLE. (Fig. 1C, D). A subset of 14 features from the 18 RBPs-associated differential genes was identified using the SVM-RFE algorithm (Fig. 1E, F). Finally, 10 overlapping feature genes between the two algorithms were selected as Hub genes (Fig. 1G).

The GSE190452 dataset provided the gene expression patterns of 28 500 cells from four TLE patient samples (Fig. S3A). nCount_RNA was positively correlated with nFeature_ RNA (Fig. S3B). Variance plots showed 25 431 genes in all cells, with red dots representing the top 2 000 highly variable genes (Fig. S3C). Seventy-one marker genes were used to identify different clusters, and the corresponding cell types were identified. Thirty independent clusters and seven cell types were identified (Fig. S3D, Fig. 1H). We conducted cellular localization analysis on the Hub gene and the results showed that CPEB3 is expressed in both excitatory and inhibitory neurons (Fig. 1I, J).

Meanwhile, based on the 10 Hub genes, we used a logistic regression algorithm to establish a diagnostic model for epilepsy classification (Fig. S4). Subsequently, we selected the top three genes in terms of AUC value for validation. RT-qPCR results showed that only CPEB3 mRNA was reduced in both hippocampus and cortex of epilepsy mice compared with control group (Fig. 1K). In summary, we speculate that downregulation of CPEB3 gene may be associated with epilepsy.

### CPEB3 expression and subcellular localization in epileptic brain tissue

**Fig. 2:**
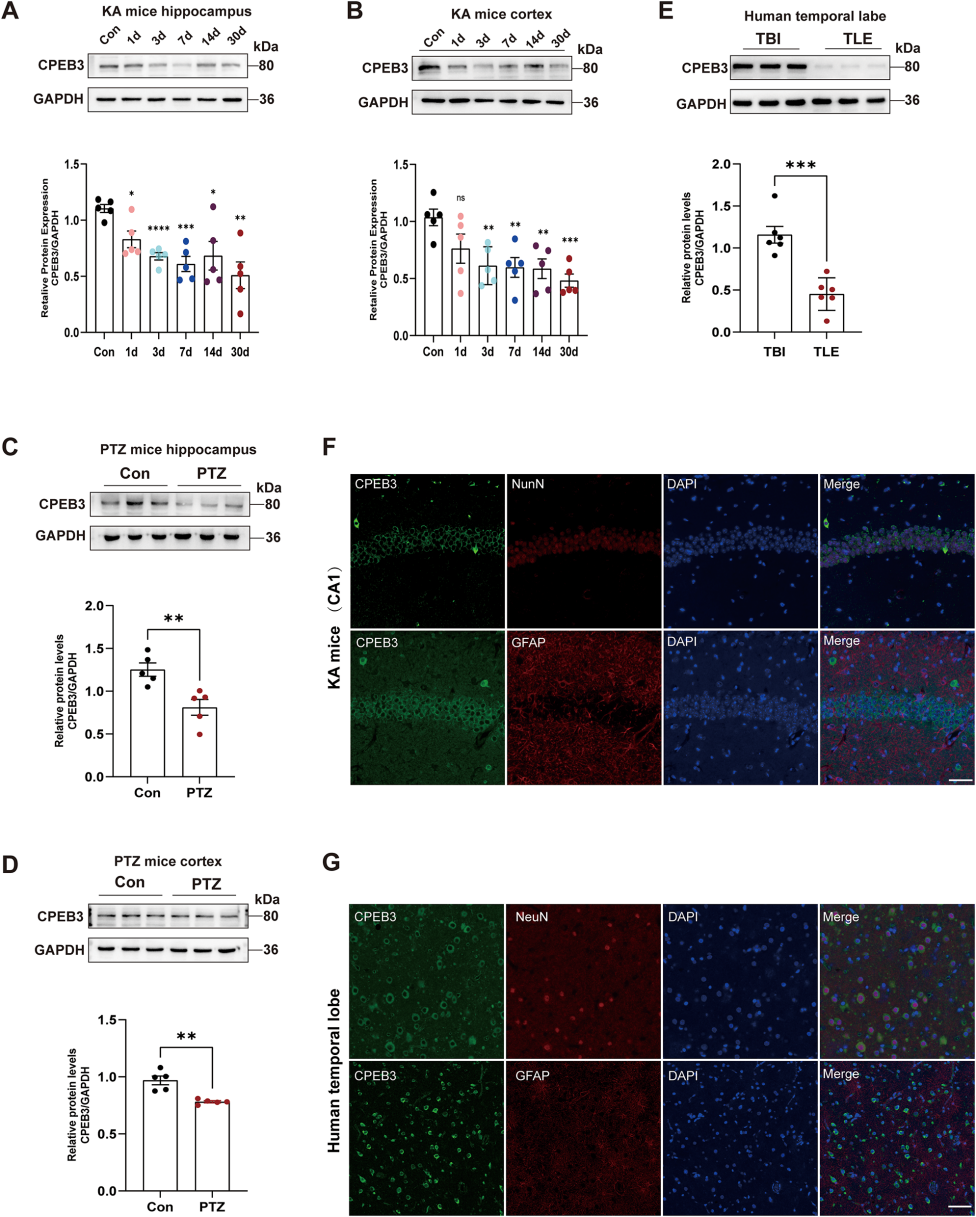
CPEB3 expression and subcellular localization in epileptic brain tissue. **A**, **B** Western blotting and quantification of CPEB3 protein expression in hippocampal (**A**) and cortex (**B**) of mice at 1, 3, 7, 14, and 30 d after KA-induced SE compared to control. (*n* = 5) **C**, **D** Western blotting and quantification of CPEB3 protein expression in hippocampal (**C**) and cortex (**D**) of PTZ-induced epileptic mice compared with control. (*n* = 5) **E** Western blotting and quantification of CPEB3 protein expression in the surgical tissue of TLE patients compared with TBI patients. (*n* = 6) **F** Immunostaining for CPEB3, NeuN and GFAP in the CA1 region of the hippocampus from the KA-induced SE mice. Scale bars = 50 μm **G** Immunostaining for CPEB3, NeuN and GFAP in the surgical tissue of TLE patients. Scale bars = 50 μm

We use WB analysis to verify the expression of CPEB3 in epileptic brain tissues. The results demonstrated that the CPEB3 expression was downregulated in the hippocampal tissue of the KA model mice than in the control group since KA-induced sustained status epilepticus (SE) at 1, 3, 7, 14, and 30 d, whereas CPEB3 did not show any significant change in the cortex at 1 d after KA injection; however, it was significantly reduced on 3, 7, 14, and 30 d (Fig. 2A, B). Meanwhile, we examined the expression of CPEB3 in PTZ model mice. We induced seizures in mice using intraperitoneal injections of PTZ at a frequency of once every two days, and extracted mouse brain tissues at the end of the 15th injection for WB. The results revealed that CPEB3 protein expression decreased in the hippocampus and cortex of PTZ model mice compared to the control group (Fig. 2C, D). Additionally, we investigated CPEB3 protein expression in TLE patients’ surgical tissues. We discovered that CPEB3 expression was substantially lower in TLE patients’ brain tissues than in TBI patients (Fig. 2E). Although our bioinformatics analysis showed that the CPEB3 gene was localized in neurons, the subcellular localization of CPEB3 remains undetermined. Therefore, we examined the distribution of CPEB3 in epileptic brain tissues using immunofluorescence staining. We found that CPEB3 was primarily co-expressed with neurons in the brain tissues of TLE patients and KA model mice (Fig. 2F, G and S5). These experimental results suggest that CPEB3 may be closely related to epilepsy.

### CPEB3 modulates seizure susceptibility and severity

**Fig. 3:**
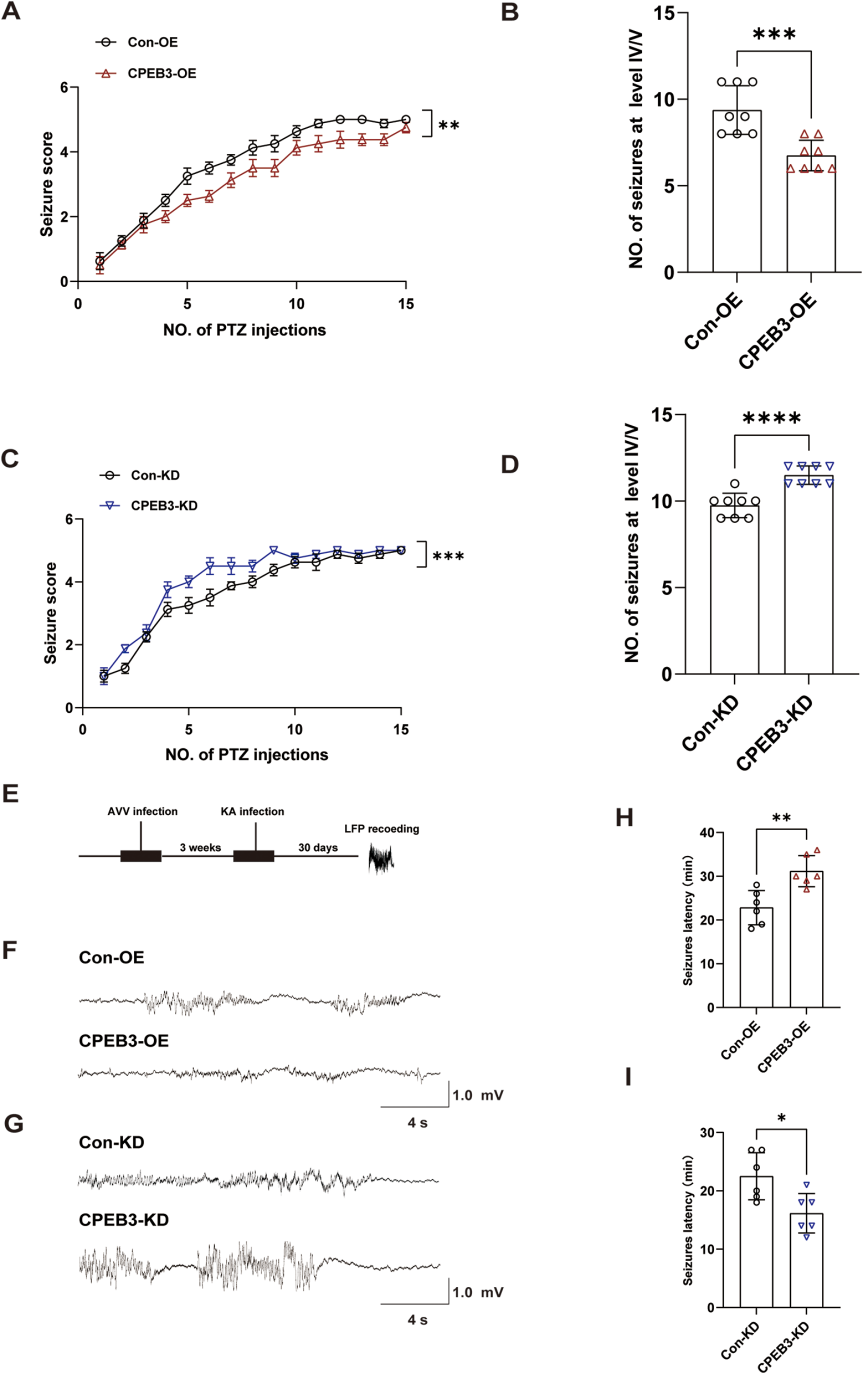
CPEB3 modulates seizure susceptibility and severity. **A** Overexpression CPEB3 reduces PTZ-induced seizure severity. (*n* = 8) **B** Overexpression CPEB3 reduces the number of seizures at level IV/V (*n* = 8). **C** Knockdown CPEB3 increases PTZ-induced seizure severity. (*n* = 8) **D** Knockdown of CPEB3 increases the number of seizures at level IV/V (*n* = 8). **E** Schematic representation of the experimental design. **F**,** G** Typical LFP showing that mice with SRSs. **H** Overexpression CPEB3 increases seizure latency (*n* = 6). **I** Knockdown CPEB3 reduces seizure latency. (*n* = 6)

To explore whether CPEB3 affects seizures, we injected AAVs into the CA1 region of the mouse hippocampus to overexpress or knockdown CPEB3. WB was used to detect the overexpression or knockdown of CPEB3 efficiency after three weeks (Fig. S6). Next, we administered an intraperitoneal injection of PTZ to induce seizures in the mice and recorded the level of each seizure. The results showed that CPEB3 overexpression significantly decreased the severity of seizures and the number of seizures at level IV/V (Fig. 3A, B). However, the severity of seizures and the number of seizures at level IV/V increased dramatically after CPEB3 knockdown (Fig. 3C, D). We recorded LFP for spontaneous recurrent seizures (SRSs) in mice, and we found that the amplitude of LFP for SRSs in overexpressing CPEB3 mice was significantly smaller than that of control mice, CPEB3 knockdown mice showed the opposite phenomenon (Fig. 3F, G). We observed the effect of CPEB3 epileptic latency in an acute epilepsy model induced using intraperitoneal injection of KA. Behavioral observation revealed that overexpression of CPEB3 significantly increased seizure latency (Fig. 3H). Knockdown of CPEB3 had the opposite effect (Fig. 3I). These results suggest that CPEB3 may modulate seizure severity and susceptibility in epileptic mice.

### CPEB3 can alter the nuclear translocation of STAT3 by inhibiting its translation

**Fig. 4:**
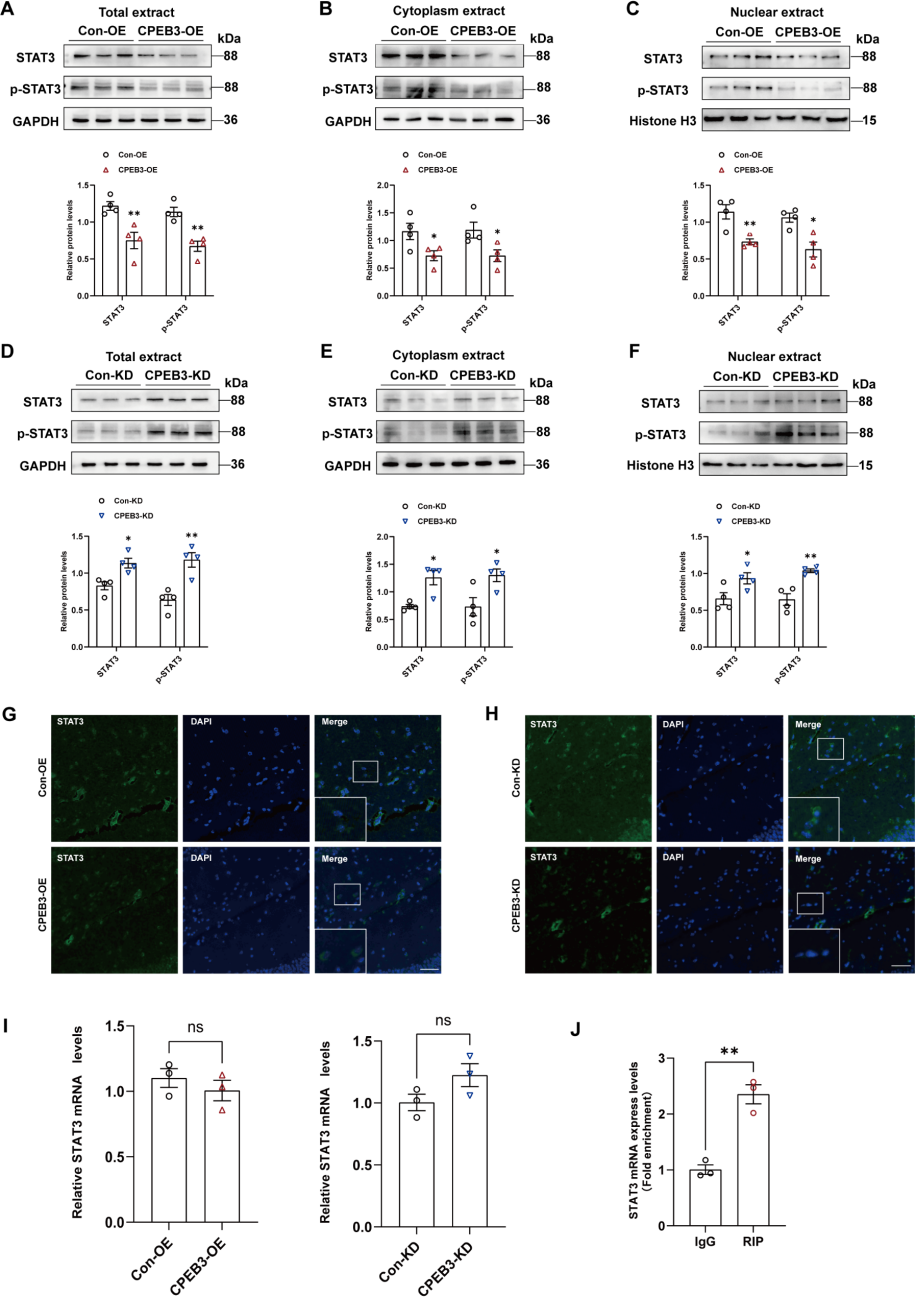
CPEB3 can alter the nuclear translocation of STAT3 by inhibiting its translation. **A**,** D** Western blotting and quantification show the expression of STAT3,p-STAT3 in hippocampal tissues of CPEB3 overexpression (**A**) or CPEB3 knockdown (**D**) mice. (*n* = 4) **B**, **E** Western blotting and quantification show the expression of STAT3,p-STAT3 in hippocampal cytoplasm extract of CPEB3 overexpression (**B**) or CPEB3 knockdown (**E**) mice. (*n* = 4) **C**, **F** Western blotting and quantification show the expression of STAT3,p-STAT3 in hippocampal nuclear extract of CPEB3 overexpression (**C**) or CPEB3 knockdown (**F**) mice. (*n* = 4) **G**, **H** Immunostaining of STAT3 in CA1 region of hippocampal tissue of CPEB3 overexpression (**G**) or CPEB3 knockdown (**H**) mice. Scale bars = 50 μm. **I** mRNA levels of STAT3 in CPEB3 overexpression or CPEB3 knockdown mice. (*n* = 3) **J** RIP experiment confirms that exogenous CPEB3 can bind to STAT3 mRNA. (*n* = 3)

To verify whether CEPB3 can regulate STAT3 expression in epilepsy, we examined changes in STAT3 in total protein lysates from mouse hippocampal tissue. We found that CPEB3 overexpression inhibited STAT3 and p-STAT3 protein expression, while STAT3 and p-STAT3 expression was elevated after CPEB3 knockdown (Fig. 4A, D). Further studies have shown that CPEB3 overexpression decreased STAT3 and p-STAT3 protein expression in the nuclear and cytoplasmic, while CPEB3 knockdown showed the opposite result (Fig. 4B, C, E, F). Immunofluorescence assay showed that nuclear STAT3 level was decreased in overexpression CPEB3 mouse hippocampal and knockdown of CPEB3 induces STAT3 into the nuclear (Fig. 4G, H). This suggests that CPEB3 inhibits the nuclear translocation of STAT3.

We used RT-qPCR to investigate the mRNA levels of STAT3 in hippocampal tissues to investigate further how CPEB3 regulates STAT3 expression. The results showed that CPEB3 did not alter STAT3 mRNA expression (Fig. 4I). Meanwhile, we verified exogenous CEPE3 expression in mouse hippocampal tissues (Fig. S7). RIP experiments showed that exogenous CPEB3 could bind to STAT3 mRNA (Fig. 4J). The above experimental results demonstrated that exogenous CPEB3 could bind to the mRNA of STAT3 to inhibit its translation, thereby altering the distribution of STAT3 in the nucleus.

### CPEB3 can regulate the expression of NMDAR subunits

**Fig. 5:**
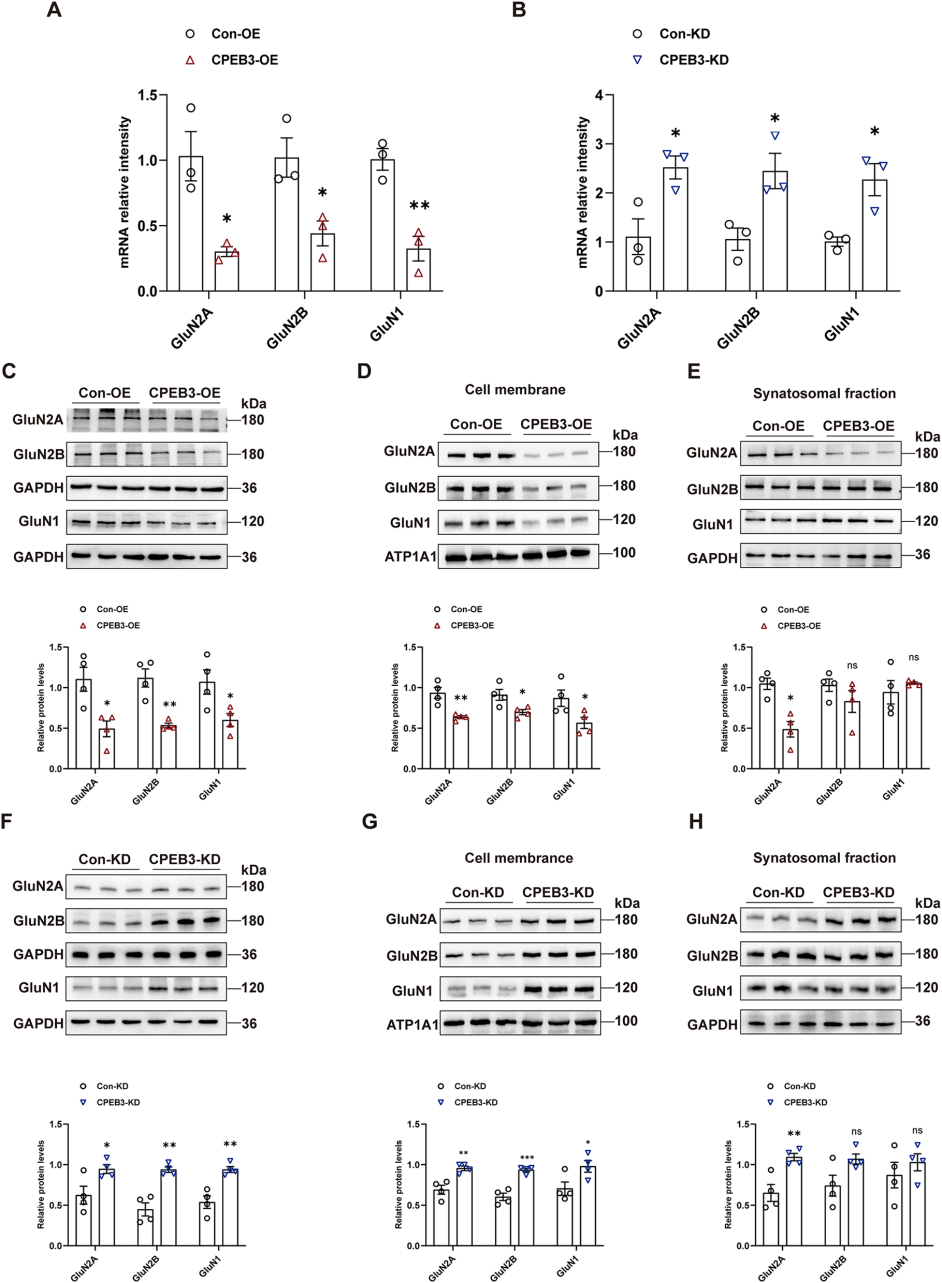
CPEB3 can regulate the expression of NMDAR subunits. **A**, **B** mRNA levels of GluN1, GluN2A, GluN2B in hippocampal of CPEB3 overexpression (**A**) or CPEB3 knockdown (**B**) mice. (*n* = 3) **C**, **F** Western blotting and quantification show the expression of GluN1, GluN2A, and GluN2B in hippocampal tissues of CPEB3 overexpression (**C**) or CPEB3 knockdown (**F**) mice. (*n* = 4) **D**, **G** Western blotting and quantification show the expression of GluN1, GluN2A, GluN2B in cell membrane of CPEB3 overexpression (**D**) or CPEB3 knockdown (**G**) mice. (*n* = 4) **E**, **H** Western blotting and quantification show the expression of GluN1, GluN2A, and GluN2B in synaptosomal fraction of CPEB3 overexpression (**E**) or CPEB3 knockdown (**H**) mice. (*n* = 4)

NMDARs overexpression causes a neuronal excitatory/inhibitory imbalance, leading to epileptic seizures. Therefore, we investigated whether CPEB3 regulates NMDARs expression in epilepsy. To verify this speculation, we examined the mRNA levels of GluN1, GluN2A, and GluN2B in hippocampal tissues. The results showed that the mRNA expression levels of the NMDAR subunit (GluN1, GluN2A, and GluN2B) in the hippocampus were regulated by CPEB3 and were negatively correlated (Fig. 5A, B). We extracted proteins from total lysates of mouse hippocampal tissues and examined NMDAR subunit expression. The results showed that the protein expression of total GluN1, GluN2A, and GluN2B in hippocampal tissues was decreased by CPEB3 overexpression and increased by CPEB3 knockdown (Fig. 5C, F). Subsequently, we extracted proteins from the cell membrane lysates of hippocampal tissues. The results demonstrated that CPEB3 had the same mechanism of negatively regulating the distribution of GluN1, GluN2A, and GluN2B in the cell membranes (Fig. 5D, G). Finally, we examined the protein expression changes of GluN1, GluN2A, and GluN2B in synaptosome lysates of hippocampal tissues. The results showed that only GluN2A is regulated by CPEB3. GluN1 and GluN2B were unaffected by CPEB3 in the synaptosome lysates (Fig. 5E, H). This suggests that CPEB3 may play a role in the pathology of epilepsy by regulating the expression of the NMDAR subunit.

### CPEB3 can alter epilepsy susceptibility and severity by inhibiting the expression of NMDARs via STAT3

**Fig. 6:**
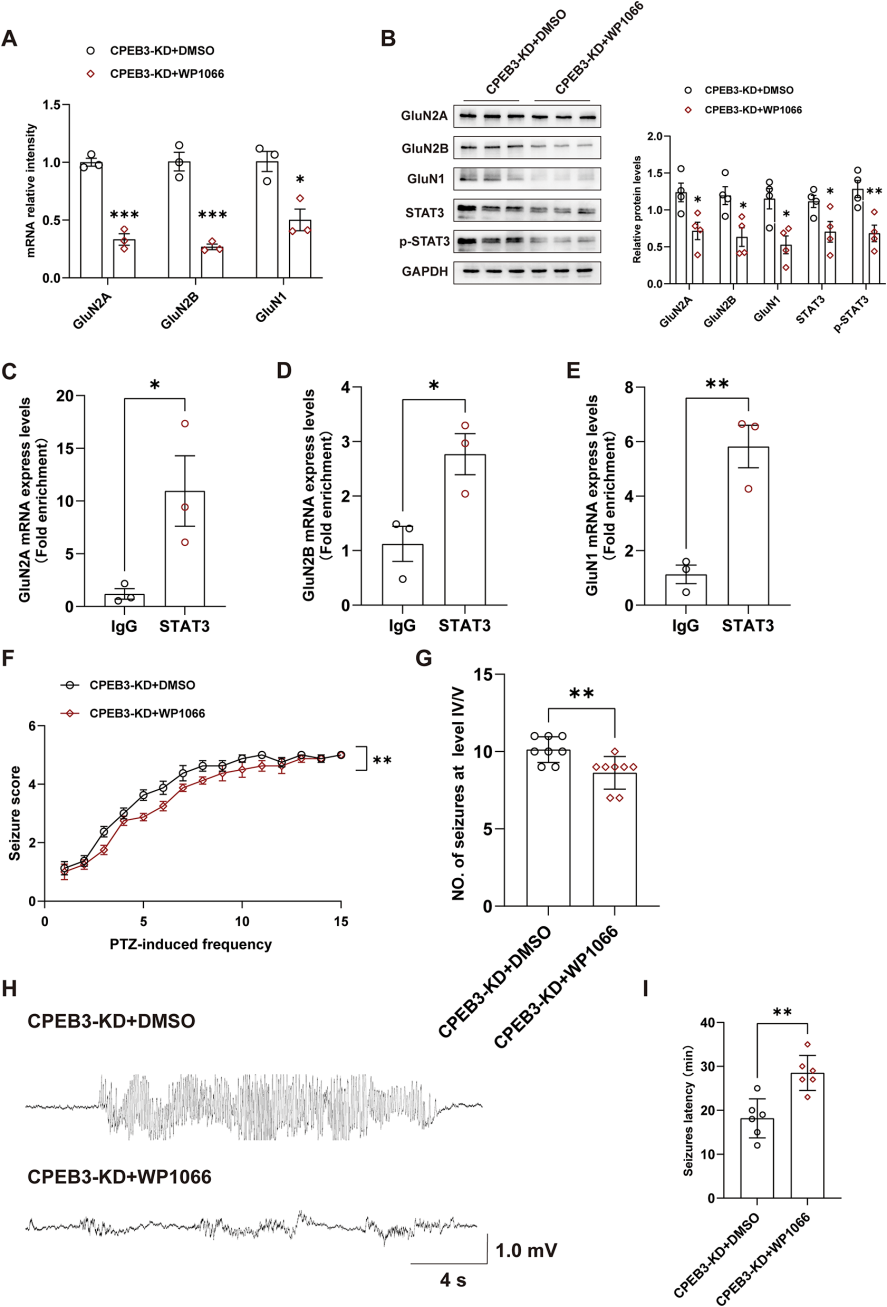
CPEB3 can alter epilepsy susceptibility and severity by inhibiting the expression of NMDARs via STAT3. **A** mRNA levels of GluN1, GluN2A, and GluN2B in hippocampal of CPEB3 knockdown mice model treated with or without WP1066. (*n* = 3) **B** Western blotting and quantification show the expression of GluN1, GluN2A, GluN2B, STAT3 and p-STAT3 in hippocampal of CPEB3 knockdown mice model treated with or without WP1066. (*n* = 4) **C-E** CHIP was used to verify the binding region of STAT3 and GluN2A (**C**), GluN2B (**D**), GluN1 (**E**) promoter. (*n* = 3) **F** Using WP1066 in knockdown CPEB3 mice reduced the severity of PTZ-induced epilepsy. (*n* = 8) **G** Using WP1066 in knockdown CPEB3 mice reduced the number of seizures at level IV/V. (*n* = 8) **H** Typical LFP showing that mice with SRSs. **I** Using WP1066 in knockdown CPEB3 mice increases seizure latency. (*n* = 6)

We injected WP1066 (a STAT3 inhibitor) intraperitoneally into knockdown CPEB3 mice and examined the protein and mRNA expression of GluN1, GluN2A, and GluN2B to verify whether CPEB3 inhibits the transcriptional activity of NMDARs via STAT3 in epilepsy. The results showed that the protein and mRNA expression of GluN1, GluN2A, and GluN2B were reduced after inhibiting STAT3 activation (Fig. 6A, B). CHIP experiments showed that STAT3 bound to the transcriptional promoters of GluN1, GluN2A, and GluN2B in mice with CPEB3 knockdown (Fig. 6C-E). Meanwhile, we found that epilepsy phenotype was reduced after STAT3 activation was inhibited (Fig. 6F-I). These results suggest that inhibition of the transcriptional activity of NMDARs by CPEB3 through STAT3 may be a novel mechanism affecting the epilepsy phenotype.

## Discussion

In this study, we investigated the mechanism of action of CPEB3 using a mice model of epilepsy. We found that CPEB3 was closely linked to epilepsy using bioinformatics analysis. CPEB3 was expressed at low levels in the brain tissues of patients with epilepsy and epileptic mouse models, and CPEB3 co-localized with neurons. CPEB3 overexpression in the hippocampus of mice reduced seizure susceptibility and severity, whereas CPEB3 knockdown increased the seizure susceptibility and severity. Further mechanistic studies revealed that exogenous overexpression of CPEB3 inhibited STAT3 translation and STAT3-mediated transcriptional activity of NMDARs, thereby suppressing NMDAR subunit expression and attenuating epilepsy phenotype in mice. Our findings provide new insights into the potential mechanisms of CPEB3 in epilepsy.

The main pathological mechanisms underlying TLE include neural death, glial cell proliferation, and altered synaptic plasticity. The development of TLE is usually accompanied by cognitive dysfunction and memory impairment. CPEB3 is a key RBP that maintains long-term memory and synaptic plasticity (Qu et al. 2020a, b; Si et al. 2010). However, whether CPEB3 plays a role in epilepsy has not yet been investigated. Our study found that the CPEB3 expression level was reduced in mice models and patients with TLE brain tissues. We also focused on the cellular localization of CPEB3 in brain tissues and found that CPEB3 was co-expressed in neurons. Next, behavioral tests revealed that CPEB3 overexpression attenuated seizures in mice. In contrast, CPEB3 knockdown exacerbated the seizure phenotype. In conclusion, CPEB3 may play a role in the pathogenesis of epilepsy, but its molecular mechanism needs to be further explored.

The JAK/STAT pathway is involved in the pathogenesis of epilepsy by affecting various biological processes, including synaptic plasticity and ferroptosis (Chen et al. 2023; Ouyang et al. 2022; Xu et al. 2024). STAT3 is an important transcription factor, mainly located in the cytoplasm in the normal state, and p-STAT3 expression increases in the activated JAK/STAT pathway. Increased p-STAT3 induces the nuclear translocation of STAT3 and increases its binding to DNA, thereby affecting target gene transcription (Liu et al. 2024). Our study found that CPEB3 inhibited STAT3 and p-STAT3 in total protein extracts, cytoplasmic and nuclear extracts. Moreover, we found that CPEB3 did not alter STAT3 mRNA expression level. Therefore, we speculated that CPEB3 inhibits the translation of STAT3 by binding to STAT3 mRNA. Subsequent RIP experiments confirmed our speculation. This suggests that CPEB3 may alters the epilepsy phenotype by inhibiting the transcriptional activity of STAT3.

NMDARs are glutamate receptors widely distributed in the central nervous system and play an important role in brain development and synaptic plasticity (Goldsmith 2019; Mony et al. 2023; Wang et al. 2021). Additional studies have shown that the expression and function of NMDARs are closely related to epilepsy (Sadeghi et al. 2021; Strehlow et al. 2019; Zhang et al. 2024). GluN1, GluN2A, and GluN2B are the main isoforms of NMDARs and are widely distributed in the cortex and hippocampus (Chen et al. 2021). Mutations in the gene encoding NMDAR subunit can lead to epilepsy (Sabo et al. 2022). In animal models of epilepsy, the expression of GluN1, GluN2A, and GluN2B subunits is increased (Postnikova et al. 2017). A nonspecific blocker of NMDARs-memantine can reduce the severity and susceptibility of epileptic mice (Gu et al. 2023). In our study, we found that CPEB3 can negatively regulate the mRNA expression levels of GluN1, GluN2A, and GluN2B in the hippocampus. The following results shown that in the hippocampus of epileptic mice, overexpression or knockdown of CPEB3 expression negatively regulated the protein expression of GluN1, GluN2A, and GluN2B in total protein lysates and cell membrane lysates in hippocampal tissues; it did not alter the protein expression of GluN1 and GluN2B in synaptosome lysates. Some studies have shown that GluN2B and GluN1 expression in the synaptosomal fractions is affected by protein interactions or post-transcriptional modifications such as phosphorylation and ubiquitination (Atkin et al. 2015; Jin et al. 2023; Rossbach et al. 2010; Zhou et al. 2021). Therefore, we speculate that these mechanisms may dominate in the synaptic, thus masking the role of CPEB3.

In this study, we demonstrated that CPEB3 inhibited the activation of STAT3 by negatively regulating the translation of STAT3 and reducing the nuclear translocation of STAT3. This suggests that CPEB3 may alter the transcription of downstream target genes by STAT3. Previous studies have disclosed that STAT3 is involved in regulating genes associated with synaptic plasticity (Tipton et al. 2023). Consequently, we speculated that CPEB3 can reduce STAT3-mediated transcription of NMDARs by inhibiting STAT3 translation in epilepsy. Next, we performed rescue experiments using WP1066, a specific inhibitor of STAT3. The results showed that the inhibition of STAT3 activation in mice with CPEB3 knockdown increased the expression of NMDARs and the mRNAs expression levels of GluN1, GluN2A, and GluN2B. CHIP experiments showed that STAT3 directly binds to the transcriptional promoters of GluN1, GluN2A, and GluN2B. Additionally, behavioral experiments demonstrated that WP1066 also reduces epileptic phenotype in mice. These results suggest that the CPEB3- STAT3- NMDAR pathway may be a novel pathway in the pathogenesis of epilepsy.

Our study has several limitations. First, Due to the lack of corresponding RIP-capable CPEB3 antibodies, we can only rely on the experimental results related to exogenous CPEB3 to infer its mechanism of action. However, the expression level, modification status, and interaction network with other molecules of endogenous proteins in cells may differ from those of exogenous overexpressed proteins, which may affect the manner and extent of the interactions between CPEB3 and target genes, making the generalizability of our experimental results limited. Second, our results suggest that CPEB3 does not regulate GluN1 and GluN2B in hippocampal synaptic lysates, and in the future, we will explore the mechanisms behind this phenomenon.

## Conclusions

In conclusion, our experimental results suggest that CPEB3 can reduce STAT3-mediated transcription of NMDARs by inhibiting STAT3 translation, which attenuates the epileptic phenotype in mice. This study provides new evidence for the role of CPEB3 in epilepsy, but further studies are needed in the future to overcome these limitations to gain a more comprehensive understanding of the mechanism of action of CPEB3.

## Electronic supplementary material

Below is the link to the electronic supplementary material.

Supplementary Material 1

Supplementary Material 2

## Data Availability

No datasets were generated or analysed during the current study.

## Abbreviations

CPEB3: Cytoplasmic polyadenylation element-binding protein 3
STAT3: Signal transducer and activator of transcription 3
p-STAT3: phosphorylated STAT3
TLE: Temporal lobe epilepsy
RBPs: RNA-binding proteins
NMDARs: N-methyl-D-aspartic acid receptors
TBI: Traumatic brain injury
GEO: Gene Expression Omnibus
KA: Kainic acid
PTZ: Pentylenetetrazole
WB: Western blotting
AAV: Adeno-associated virus
LTP: Local field potentials
RT-qPCR: Real-time quantitative polymerase chain reaction
RIP: RNA immunoprecipitation
CHIP: Chromatin immunoprecipitation
SE: Status epilepticus
SRSs: Spontaneous recurrent seizures
