# Supplementary material for: CPEB3 can regulate seizure susceptibility by inhibiting the transcriptional activity of STAT3 on NMDARs expression
Source: Mol Med. 2025 Feb 24;31:77. doi: 10.1186/s10020-025-01136-2 (PMC11852879; doi:10.1186/s10020-025-01136-2)
Supplement: Supplementary file 1 — Supplementary Material 1 [file 10020_2025_1136_MOESM1_ESM.docx]

**Supplementary Materia**

**MATERIALS AND METHODS**

**Gene Expression Omnibus (GEO) dataset collection**

Datasets GSE63808 and GSE29378 were downloaded from the GEO database. Dataset GSE63808 contains 129 hippocampal tissue samples from patients with chronic TLE, while dataset GSE29378 contains 32 autopsy normal hippocampal tissue samples. GSE63808 and GSE29378 were merged using the R software in the SilicoMerging package, and batch effects were removed using Johnson WE et al(Johnson et al. 2007; Taminau et al. 2012). After removing batch effects, the final matrix was obtained, containing 129 epilepsy samples and 32 control samples for subsequent analyses.

**Extraction of RBPs-related genes**

The set of RBPs-related genes was extracted from the EuRBPDB database, a comprehensive and user-friendly database of eukaryotic RBPs. 2 994 human RBPs-related genes were downloaded from the EuRBPDB database, and gene expression in the merged dataset was extracted to construct expression profile matrices of RBPs-related genes for subsequent analyses.

**Differential expression analysis**

To find RPBs-related genes that were differently expressed across epilepsy and control groups, a differential analysis of RBPs-related gene expression profile matrices was performed. The significance criterion was set at *p* < 0.05. RBPs-related differentially expressed genes were visualized and analyzed by constructing heatmaps using the heatmap package and volcano plots using the ggplot2 package. Finally, the Gene Ontology (GO) and The Kyoto Encyclopedia of Genes and Genomes (KEGG) analyses were performed on the differentially expressed genes.

**Single-cell analysis**

The single-cell data was selected from the GEO database (GSE190452) containing four patients with TLE and analyzed using the "Seurat" and "CellChat" software packages(Jin et al. 2021; Satija et al. 2015). High-quality data were kept by removing low-quality cells, and cells expressing less than 200 or more than 6 000 genes as well as those with a mitochondrial gene ratio of more than 10% were eliminated. Finally, cells with a gene count of greater than 200 000 were obtained. Finally, 28 500 cells, including four temporal lobe epilepsies, were identified. The top 20 principal components (PCs) based on the top 2 000 highly variable genes were extracted using principal component analysis (PCA) after the gene expression of the included cells was normalized using the "NormalizeData" function. These PCs were then saved for additional analysis using the "FindVariableFeatures" function. For unsupervised and unbiased clustering, cell subgroups, "FindNeighbors," "FindClusters" (resolution = 0.5), and "RunTSNE" functions were applied.

**Screening of Hub genes and construction of diagnostic model**

The Hub genes were screened using LASSO and SVM-RFE to find the aforementioned RBPs-related differentially expressed genes. Using a logistic regression technique, a novel epilepsy diagnostic model was built based on these hub genes. The Area Under Curve (AUC) was used to assess the accuracy of Hub genes and the logistic regression model. In the end, the process described above was visually analyzed using the ggplot2 package (version 3.3.5) and the qROC tool (version 1.18.0).

**REFERENCES**

Jin S, Guerrero-Juarez CF, Zhang L, Chang I, Ramos R, Kuan CH, et al. Inference and analysis of cell-cell communication using CellChat. Nat Commun. 2021;12(1):1088.

Johnson WE, Li C, Rabinovic A. Adjusting batch effects in microarray expression data using empirical Bayes methods. Biostatistics. 2007;8(1):118-27.

Satija R, Farrell JA, Gennert D, Schier AF, Regev A. Spatial reconstruction of single-cell gene expression data. Nat Biotechnol. 2015;33(5):495-502.

Taminau J, Meganck S, Lazar C, Steenhoff D, Coletta A, Molter C, et al. Unlocking the potential of publicly available microarray data using inSilicoDb and inSilicoMerging R/Bioconductor packages. BMC Bioinformatics. 2012;13:335.

**Supplementary figures and** **figure legends**

**Figure.S1**

**
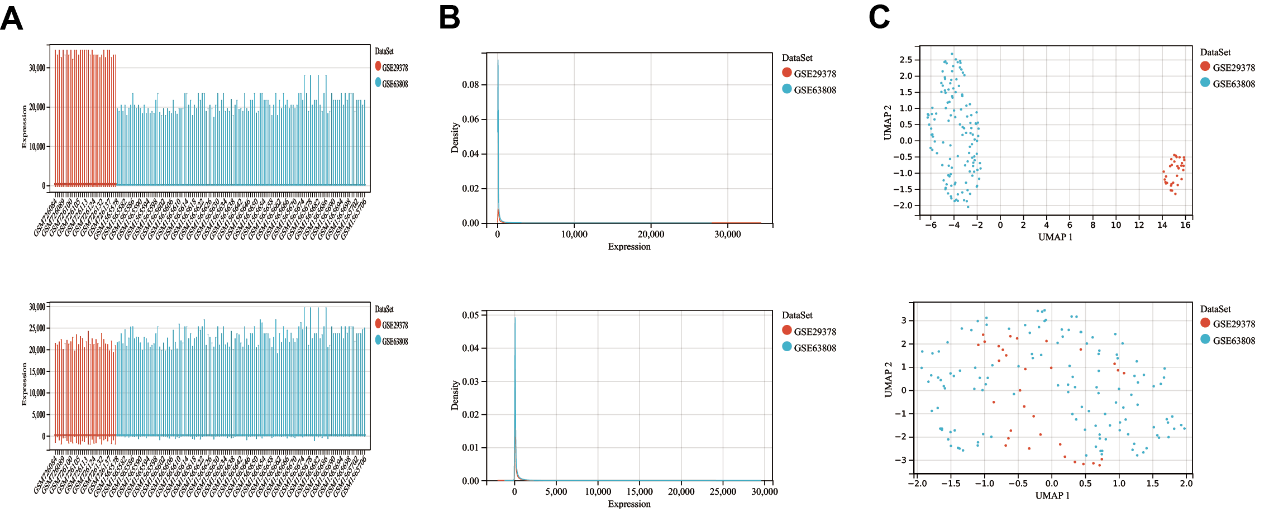
**

**Fig.S1 Combined data are processed to remove batch effects. A** Boxplots before and after removing batch effects**. B** Density plots before and after removing batch effects. **C** UMAP plots before and after removing batch effects.

**Figure.S2**


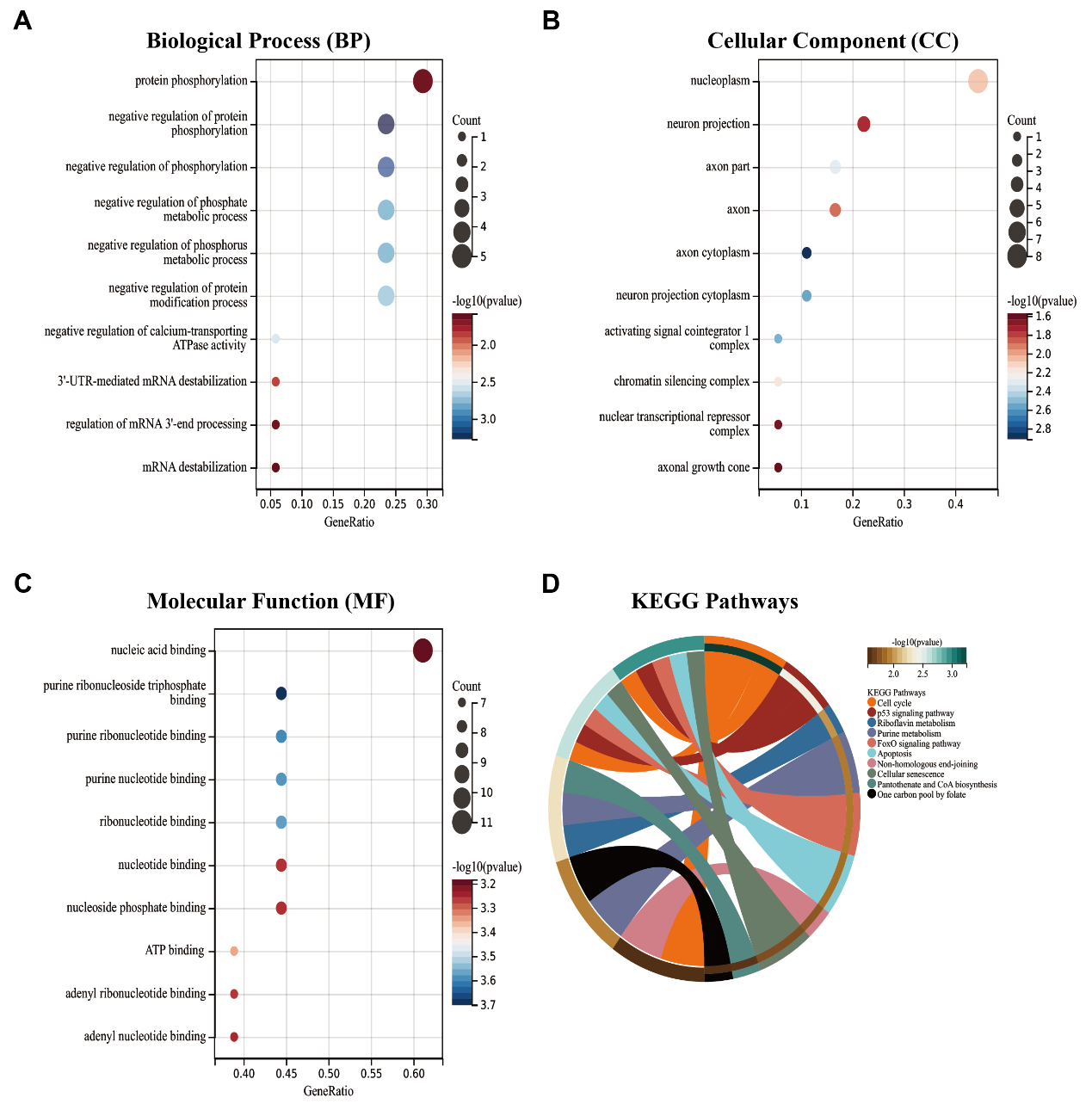


**Fig.S2 The results of GO and KEGG enrichment analysis of 18 RBPs-related differentially expressed genes are shown in the bubble chart and circle plots. A** The top 10 biological processes (BP). **B** The top 10 cellular components(CC)**. C** The top 10 molecular functions (MF). **D** The top 10 KEGG pathways.

**Figure.S3**


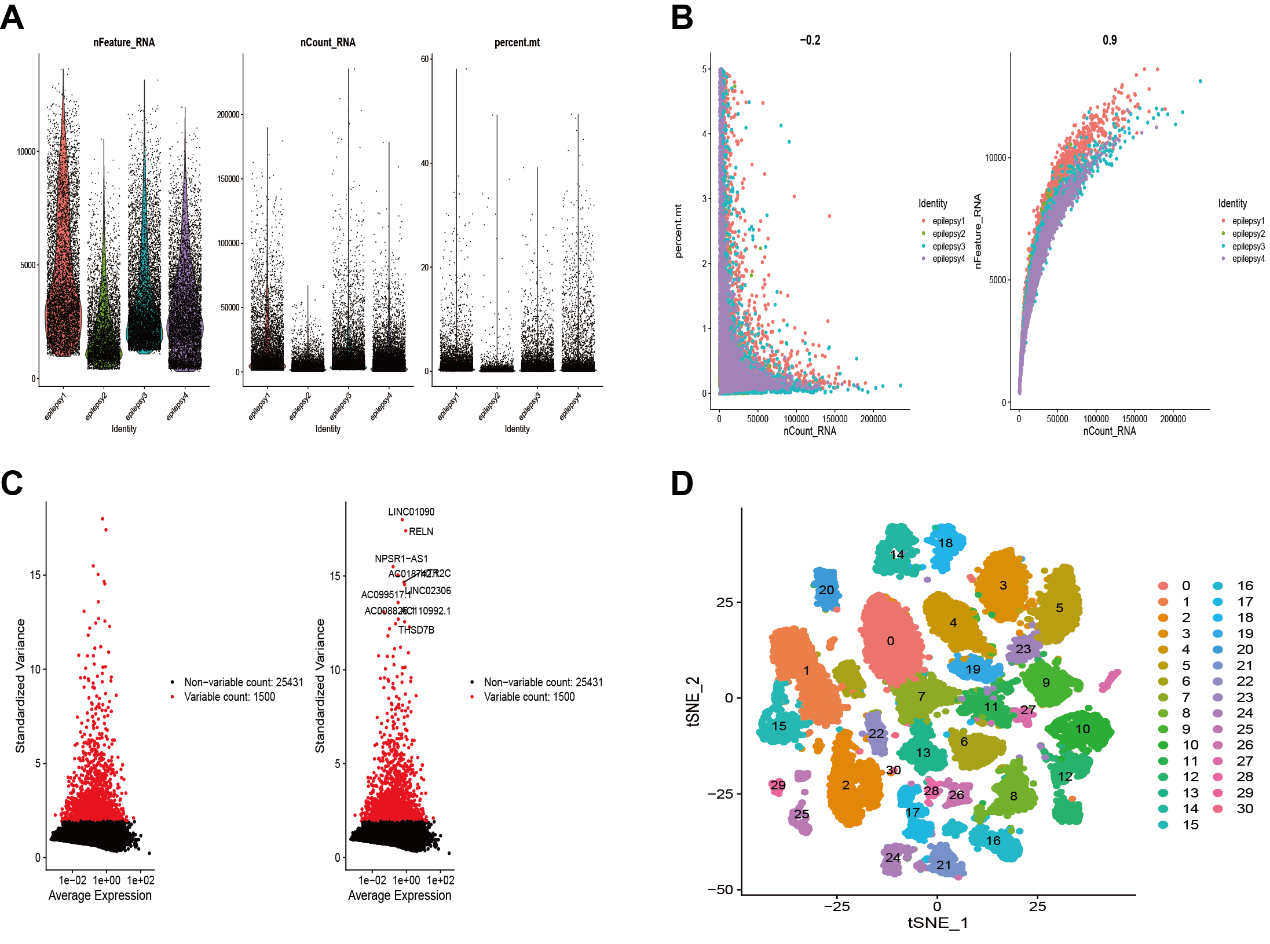


**Fig.S3** **Data processing for single-cell analysis. A** Number of genes per cell (nFeature_RNA), number of unique molecular identifications (UMI) per cell (nCount_RNA), and percentage of mitochondrial genes per cell (percent. mt) in single-cell RNA-seq data. **B** Correlation between nCount_RNA and nFeature_RNA. Correlation between percent. mt and nFeature_RNA. **C** The variance plot shows 25,341 genes in all cells, with red dots representing the top 2,000 highly variable genes. **D** Cells were divided into 30 separate clusters.

**Figure.S4**


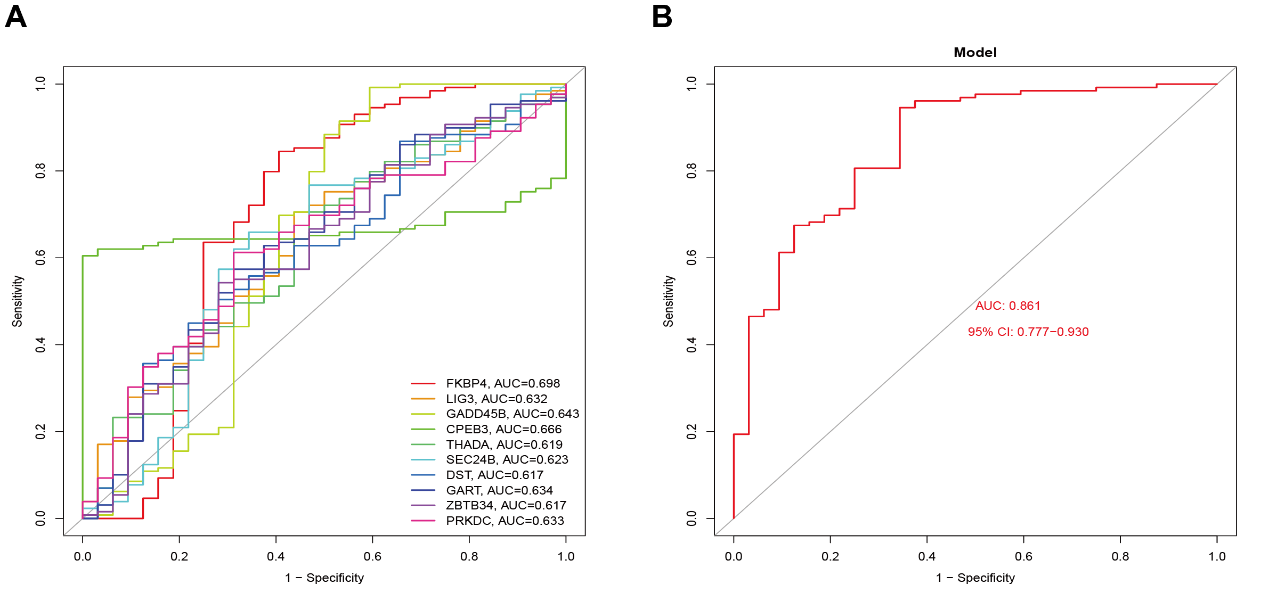


**Fig.S4 Logistic regression model construction and ROC curve validation**. **A** ROC curve for the Hub gene. ( FKBP4 (AUC = 0.698)、LIG3 (AUC =0.632)、GADD45B (AUC = 0.643)、CPEB3 (AUC = 0.666)、THADA (AUC =0.619)、SEC24B (AUC = 0.623)、DST (AUC = 0.617)、GART (AUC =0.634)、ZBTB34 (AUC = 0.617)、PRKDC (AUC = 0.633)) **B** ROC curve of the model.

**Figure.S5**


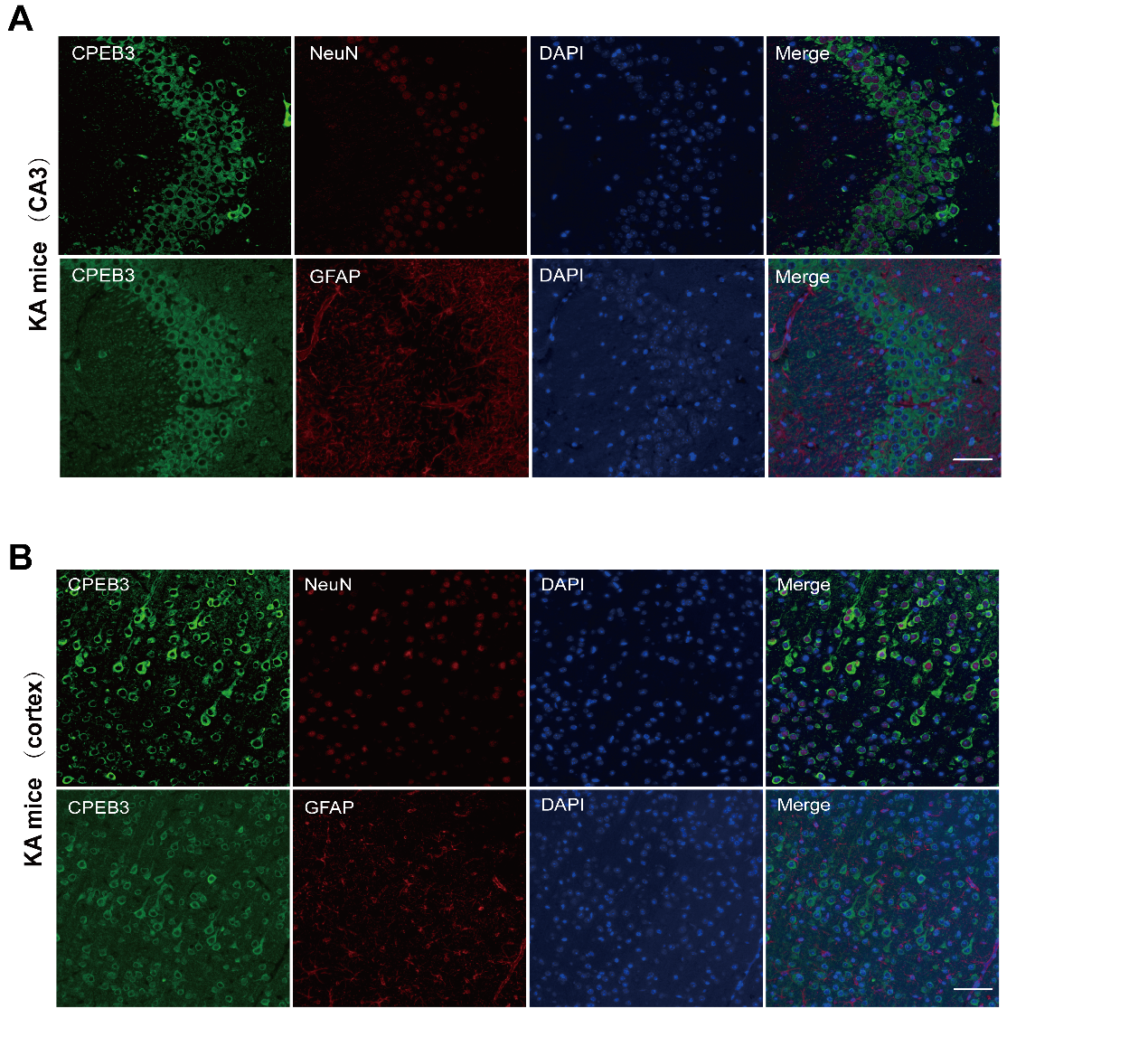


**Fig.S5 Immunostaining for CPEB3 in epileptic brain tissues. A** Immunostaining for CPEB3, NeuN, and GFAP in the CA3 region of the hippocampus from the KA-induced SE mice. Scale bars = 50 μm **B** Immunostaining for CPEB3, NeuN, and GFAP in the cortex of the hippocampus from the KA-induced SE mice. Scale bars = 50 μm

**Figure.S6**


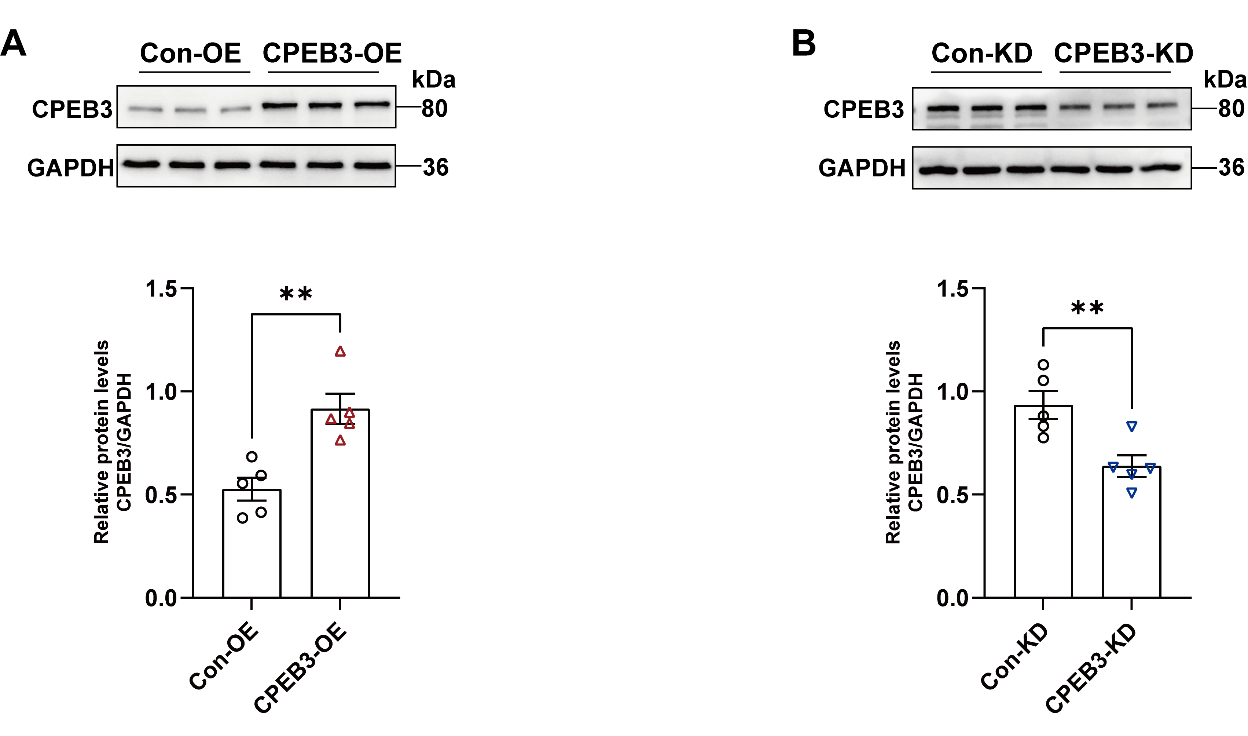


**Fig.S6 Validation of CPEB3 overexpression or knockdown in the hippocampus. A, B** Western blotting and quantification of CPEB3 protein levels in mice hippocampal tissue 21 d after AAVs infection. (n=5)

**Figure.S7**


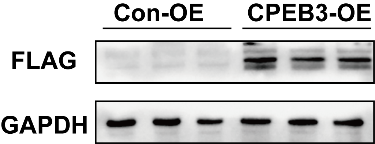


**Fig.S7 Western blotting for exogenous CPEB3.**

**Table S1.** **Clinical characteristics of patients in epilepsy and control groups**

| Case | Gender | Age  (years) | Duration of epilepsy (years) | Operative side | Other neurological disorders |
| --- | --- | --- | --- | --- | --- |
| E1 | Male | 27 | 15 | Left | No |
| E2 | Male | 36 | 7 | Right | No |
| E3 | Female | 29 | 13 | Left | No |
| E4 | Female | 22 | 9 | Left | No |
| E5 | Female | 40 | 10 | Right | No |
| E6 | Male | 19 | 11 | Right | No |
| C1 | Female | 23 | 0 | Left | No |
| C2 | Female | 45 | 0 | Left | No |
| C3 | Male | 37 | 0 | Right | No |
| C4 | Male | 50 | 0 | Left | No |
| C5 | Male | 22 | 0 | Right | No |
| C6 | Female | 16 | 0 | Right | No |

E=epilepsy; C=control.

**Table S2. Antibody details.**

| **Antibody** | **Source** | **Catalog Number** | **Species** | **Applications** |
| --- | --- | --- | --- | --- |
| CPEB3 | Proteintech | 12669-1-AP | Rabbit | WB (1:1000)  IF (1:100) |
| GAPDH | Proteintech | 10494-1-AP | Rabbit | WB (1:5000) |
| NeuN | Proteintech | 66836-1-Ig | Mouse | IF (1:100) |
| GFAP | Proteintech | 60190-1-lg | Mouse | IF (1:100) |
| GluN1 | Proteintech | 27676-1-AP | Rabbit | WB (1:2000) |
| GluN2A | Proteintech | 28571-1-AP | Rabbit | WB (1:2000) |
| GluN2B | Proteintech | 21920-1-AP | Rabbit | WB (1:2000) |
| ATP1A1 | Proteintech | 14418-1-AP | Rabbit | WB(1:10000) |
| STAT3 | Proteintech | 10253-2-AP | Rabbit | IF(1:100) |
| STAT3 | Proteintech | 60199-1-Ig | Mouse | WB (1:1000) |
| p-STAT3 (Tyr705) | Zenbio | 381552 | Rabbit | WB (1:1000) |
| Flag tag | Proteintech | 20543-1-AP | Rabbit | WB (1:2000) |
| Histone H3 | PTMBio | PTM-6600 | Rabbit | WB (1:1000) |

**Table S3. Sequences of qPCR primers.**

| **Gene** | **Forward primer sequence** | **Reverse primer sequence** |
| --- | --- | --- |
| FKBP4 | AGCAACAAAGCCGCCAAGACC | GCTGCCACTTCTGCCTTCACC |
| GADD45B | CTGGTGGCGAGCGACAACG | ACGGTGAGGCGATCCTGACG |
| CPEB3 | CGTGGGCGTGGGTGTAGG | GCGATCACATTGCTGGAGAAGG |
| GluN2A | CTTGTGGTGATCGTGCTGAATAAGG | ATGCTGAGGTGGTTGTCATCTGG |
| GluN2B | AGCGACCTGTACGGCAAGTTC | ATAGGTGACAGTATGCGTGGAGATG |
| GluN1 | CGGAGGCAGGTGGAGTTGAG | ATGAAGGCATGGAGCTTGTTGTC |
| STAT3 | AATCTCAACTTCAGACCCGCCAAC | GCTCCACGATCCTCTCCTCCAG |
| GAPDH | GCGAAGAAAACCGCATCAC | CACACCTCACATCACCACGTC |
| GluN2A（chip） | ACAGCAGCACAGTACAAGACA | TATGGGAAGAGAGTGTCACATGC |
| GluN2B（chip） | GCTGGGTTAGTCAGTGCTGT | TGTGTGATATTTTCCGGCTGC |
| GluN1  （chip） | GGCTGTACTTTACATGGCAGG | CTGCTTCCTCTCTTTGGCTGT |

**Table S4. Differently expressed genes.**

| **Gene** | **conMean** | **treatMean** | **pvalue** | **Type** |
| --- | --- | --- | --- | --- |
| FKBP4 | 8.037404031 | 8.503879496 | 0.000542679 | Up |
| LIG3 | 7.033556938 | 6.99648614 | 0.020845178 | Down |
| BAZ2A | 6.852393094 | 6.916350434 | 0.033285226 | Up |
| GADD45B | 7.6255085 | 7.846503031 | 0.012669453 | Up |
| ZC3H14 | 6.966831219 | 6.933257085 | 0.016790552 | Down |
| CPEB3 | 7.485408188 | 7.406578922 | 0.003686319 | Down |
| CHORDC1 | 6.909617031 | 7.027169969 | 0.015302026 | Up |
| ASCC3 | 7.136073344 | 7.096542721 | 0.006663573 | Down |
| THADA | 7.142913375 | 7.108955845 | 0.037339676 | Down |
| GADD45G | 8.289950406 | 8.089827302 | 0.015302026 | Down |
| SEC24B | 6.982253125 | 6.948254876 | 0.031570545 | Down |
| DST | 6.773868844 | 6.754189411 | 0.040546232 | Down |
| EIF5B | 8.14880525 | 8.301790783 | 0.045333825 | Up |
| GART | 7.293846125 | 7.255803047 | 0.019044156 | Down |
| PBRM1 | 6.914887156 | 6.882884783 | 0.049597497 | Down |
| ZBTB34 | 7.150559906 | 7.116841953 | 0.041807393 | Down |
| ENPP1 | 6.931949 | 7.044954434 | 0.048622024 | Up |
| PRKDC | 7.324279469 | 7.284199062 | 0.019926911 | Down |
