# Supplementary material for: CPEB3 can regulate seizure susceptibility by inhibiting the transcriptional activity of STAT3 on NMDARs expression
Source: Mol Med. 2025 Feb 24;31:77. doi: 10.1186/s10020-025-01136-2 (PMC11852879; doi:10.1186/s10020-025-01136-2)
Supplement: Supplementary file 2 — Supplementary Material 2 [file 10020_2025_1136_MOESM2_ESM.docx]

Fig.2A-CPEB3





Fig.2A-GAPDH





Fig.2B-CPEB3





Fig.2B-GAPDH





Fig.2C-CPEB3





Fig.2C-GAPDH





Fig.2D-CPEB3





Fig.2D-GAPDH





Fig.2E-CPEB3





Fig.2E-GAPDH





Fig.4A-STAT3





Fig.4A-p-STAT3





Fig.4A-GAPDH





Fig.4B-STAT3





Fig.4B-p-STAT3





Fig.4B-GAPDH





Fig.4C-STAT3





Fig.4C-p-STAT3





Fig.4C-Histone H3





Fig.4D-STAT3





Fig.4D-p-STAT3





Fig.4D-GAPDH





Fig.4E-STAT3





Fig.4E-p-STAT3





Fig.4E-GAPDH





Fig.4F-STAT3





Fig.4F-p-STAT3





Fig.4F-Histone H3





Fig.5C-GluN2A





Fig.5C-GluN2B





Fig.5C-GAPDH





Fig.5C-GluN1





Fig.5C-GAPDH





Fig.5D-GluN2A





Fig.5D-GluN2B





Fig.5D-GluN1





Fig.5D-ATP1A1





Fig.5E-GluN2A





Fig.5E-GluN2B





Fig.5E-GluN1





Fig.5E-GAPDH





Fig.5F-GluN2A





Fig.5F-GluN2B





Fig.5F-GAPDH





Fig.5F-GluN1





Fig.5F-GAPDH





Fig.5G-GluN2A





Fig.5G-GluN2B





Fig.5G-GluN1





Fig.5G-ATP1A1





Fig.5H-GluN2A





Fig.5H-GluN2B





Fig.5H-GluN1





Fig.5H-GAPDH





Fig.6B-GluN2A





Fig.6B-GluN2B





Fig.6B-GluN1





Fig.6B-STAT3





Fig.6B-p-STAT3





Fig.6B-GAPDH





Fig.S6A-CPEB3





Fig.S6A-GAPDH





Fig.S6B-CPEB3





Fig.S6B-GAPDH





Fig.S7-FLAG





Fig.S7-GAPDH
